# Suramin-Induced Neurotoxicity: Preclinical Models and Neuroprotective Strategies

**DOI:** 10.3390/molecules23020346

**Published:** 2018-02-07

**Authors:** David von der Ahe, Petra Huehnchen, Mustafa Balkaya, Sarah Peruzzaro, Matthias Endres, Wolfgang Boehmerle

**Affiliations:** 1 Charité—Universitätsmedizin Berlin, Corporate Member of Freie Universität Berlin, Humboldt-Universität zu Berlin, and Berlin Institute of Health, Klinik und Hochschulambulanz für Neurologie, Chariteplatz 1, 10117 Berlin, Germany; david.von-der-ahe@charite.de (D.v.d.A.); petra.huehnchen@charite.de (P.H.); wolfgang.boehmerle@charite.de (W.B.); 2 Charité—Universitätsmedizin Berlin, Corporate Member of Freie Universität Berlin, Humboldt-Universität zu Berlin, and Berlin Institute of Health, Cluster of Excellence NeuroCure, 10117 Berlin, Germany; 3 Berlin Institute of Health, Anna-Louisa-Karsch 2, 10178 Berlin, Germany; 4 Burke-Cornell Medical Research Institute, White Plains, NY 10605, USA; mgbalkaya@yahoo.com; 5 Field Neurosciences Institute Laboratory for Restorative Neurology, Central Michigan University, Mt. Pleasant, MI 48859, USA; stperuzzaro@gmail.com; 6 Charité—Universitätsmedizin Berlin, Corporate Member of Freie Universität Berlin, Humboldt-Universität zu Berlin, and Berlin Institute of Health, Center for Stroke Resarch Berlin, 10117 Berlin, Germany; 7 German Center for Neurodegenerative Diseases (DZNE), 10117 Berlin, Germany; 8 German Center for Cardiovascular Research (DZHK), Partner Site Berlin, 10117 Berlin, Germany

**Keywords:** suramin, calcium, neuroprotection, voltage-gated calcium channels, TRP channels

## Abstract

Suramin is a trypan blue analogon originally developed to treat protozoan infections, which was found to have diverse antitumor effects. One of the most severe side effects in clinical trials was the development of a peripheral sensory-motor polyneuropathy. In this study, we aimed to investigate suramin-induced neuropathy with a focus on calcium (Ca^2+^) homeostasis as a potential pathomechanism. Adult C57Bl/6 mice treated with a single injection of 250 mg/kg bodyweight suramin developed locomotor and sensory deficits, which were confirmed by electrophysiological measurements showing a predominantly sensory axonal-demyelinating polyneuropathy. In a next step, we used cultured dorsal root ganglia neurons (DRGN) as an in vitro cell model to further investigate underlying pathomechanisms. Cell viability of DRGN was significantly decreased after 24-hour suramin treatment with a calculated IC_50_ of 283 µM. We detected a suramin-induced Ca^2+^ influx into DRGN from the extracellular space, which could be reduced with the voltage-gated calcium channel (VGCC) inhibitor nimodipine. Co-incubation of suramin and nimodipine partially improved cell viability of DRGN after suramin exposure. In summary, we describe suramin-induced neurotoxic effects on DRGN as well as potentially neuroprotective agents targeting intracellular Ca^2+^ dyshomeostasis.

## 1. Introduction

Suramin is a water-soluble polysulfonated naphtylurea. It was first synthesized in 1916 as a colorless derivate of trypan dyes for the treatment of African trypanosomiasis (reviewed by [1]). Later on it was found that suramin had an effect against the Human-Immunodeficiency Virus [2] and that non-Hodgkin lymphomas associated with this virus were sensitive to suramin treatment [3]. Further investigation revealed broad antitumor activity of suramin and a wide variety of mechanisms of action were described. Among others, it was shown that suramin inhibits several different enzymes and growth factors, which may account for the antiretroviral, antiprotozoal and antitumor activity. In different tumor entities suramin was proposed to bind to a range of growth factors such as the vascular endothelial growth factor or the epidermal growth factor [1]. Subsequently, suramin was tested in clinical trials of different metastatic tumor entities with emphasis on metastatic prostate cancer [4,5,6,7]. In line with the seemingly unspecific mode of action of suramin, the clinical application revealed several side effects such as coagulopathy, adrenal insufficiency, nephrotoxicity or hepatotoxicity [1,6]. One of the most frequently observed dose limiting side effects, however, is a toxic peripheral polyneuropathy, which occurs in 40–79% of suramin treated patients [6,8,9]. As suramin is a charged molecule and does not cross the blood brain barrier it exerts neurotoxic effects in the peripheral nervous system only [10]. Due to the potentially severe side effects, clinical use of suramin at the time of writing is limited to treatment of African trypanosomiasis and as an orphan drug for (metastatic) hormone refractory prostate cancer. Suramin-induced peripheral neuropathy most frequently shows as sensory-motor axonal neuropathy with sensory symptoms such as hypoesthesia, paresthesia, dysesthesia, hypoalgesia or pallhypesthesia [9,11]. The incidence of a demyelinating Guillian-Barré like syndrome under suramin treatment was reported less frequently [12]. Incidence and severity of suramin-induced neuropathy are influenced by plasma levels exceeding 350 µg/mL, the cumulative suramin dose and the duration of chemotherapy [12,13]. A variety of different pathomechanisms was described: The inhibition of lysosomal enzymes by suramin was proposed to cause an experimental form of gangliosidosis with intracellular lamellar inclusion bodies in neurons and Schwann cells [14,15]. Different experimental studies suggested the involvement of nerve growth factor and a binding affinity of suramin to the nerve growth factor receptor to account for the neurotoxic effect [16,17]. Furthermore, Sun et al. described that suramin leads to a disturbance of the intracellular calcium (Ca^2+^) homoeostasis in dorsal root ganglia neurons (DRGN) [18]. Intriguingly, other chemotherapeutic agents such as paclitaxel and salinomycin were also found to cause a peripheral neuropathy via a disturbance of intracellular Ca^2+^ levels [19,20]. Thus, it appears that the disruption of the intracellular Ca^2+^ homeostasis might be a common pathomechanism in chemotherapy-induced polyneuropathies. The aim of this study was thus to establish preclinical models of suramin-induced neurotoxicity in order to further elucidate the underlying pathomechanism with an emphasis on Ca^2+^ signaling and to identify potential molecular targets suitable for neuroprotection. We observed that suramin induces neurotoxicity in vitro and in vivo. Calcium influx into DRGN from the extracellular space seems to partially mediate suramin-induced neurotoxicity, as Nimodipine, an L-Type voltage-gated calcium channel (VGCC) inhibitor, showed limited neuroprotective effects.

## 2. Results

### 2.1. Suramin Induces a Sensory Axonal-Demyelinating Polyneuropathy in C57Bl/6 Mice

**Figure 1:**
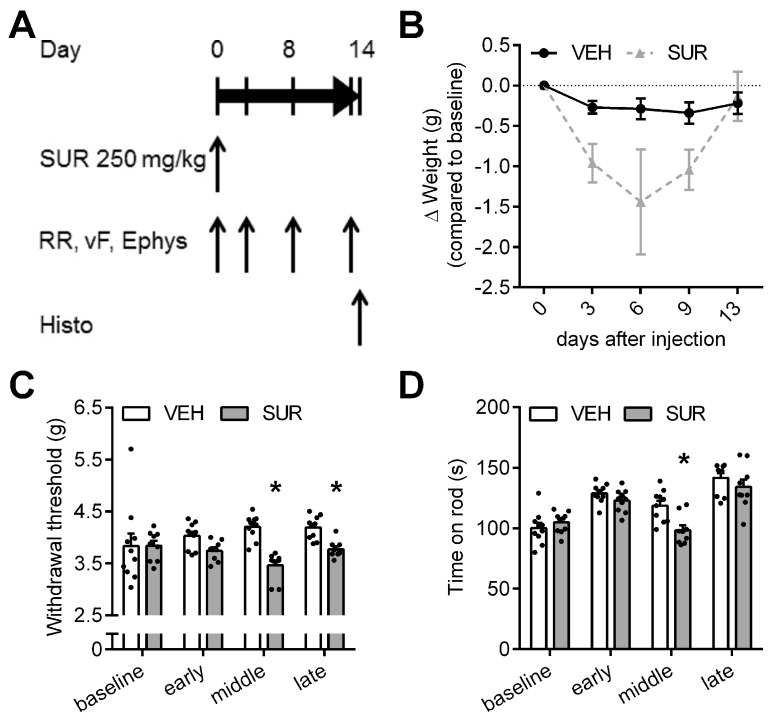

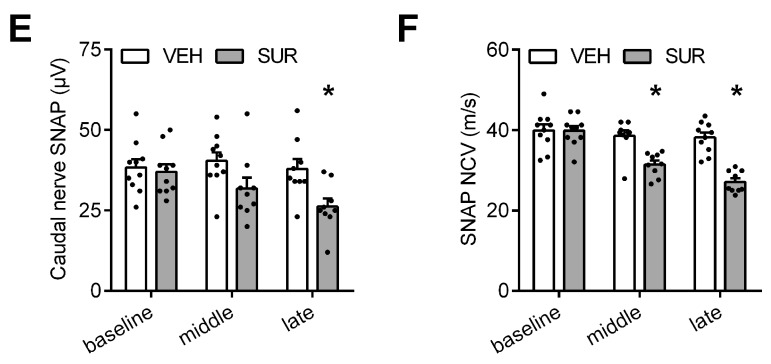
Mouse model of suramin-induced sensory-motor polyneuropathy. (**A**) Schematic outline of the experiment: Adult C57Bl/6 mice received a single intraperitoneal injection of 250 mg/kg bodyweight suramin (SUR) or vehicle (VEH) on day 0. Mice were tested on the rotarod (RR) and with the von Frey (vF) test for locomotor deficits and alterations of mechanical withdrawal threshold four times. Electropysiological (Ephys) measurements were obtained on day 0, 8 and 13; (**B**) SUR treated mice showed a decline in body weight, but recovered by day 13. One animal had to be sacrificed due to weight loss >20%; (**C**) SUR application resulted in a decrease of the mechanical withdrawal threshold and (**D**) locomotor function, which was most pronounced on day 8; (**E**) SUR treated mice showed a decline of the sensory nerve action potential (SNAP) amplitude as well as the (**F**) SNAP nerve conduction velocity (NCV), indicative of an axonal-demyelinating neuropathy. Group sizes: *n* = 10 (vehicle), *n* = 9–10 (suramin), * *p* < 0.05.

We previously characterized the behavioral and histological phenotype of chemotherapy-induced polyneuropathies in C57Bl/6 mice after application of paclitaxel, vincristine, cisplatin and bortezomib, respectively [21]. In order to establish a mouse model of a suramin-induced polyneuropathy, we adapted a previously published protocol. Russell et al. had shown that a single intraperitoneal injection of 500 mg/kg bodyweight suramin can induce chemotherapy-induced polyneuropathy in rats [15]. In mice, the published LD_50_ of intraperitoneal suramin application is 750 mg/kg bodyweight [22]. To avoid unspecific general side effects, we lowered the dose to a single intraperitoneal injection of 250 mg/kg bodyweight suramin. Mice were tested for sensory and motor deficits on days 3, 8 and 13 after suramin application (Figure 1A). Suramin treatment resulted in a steady decline in bodyweight, which reached its maximum on day 6 with −1.44 g ± 0.65 g compared to baseline (not significant, Kruskal-Wallis test; Figure 1B). Afterwards, mice regained bodyweight and reached control levels by the end of the experiment. One animal, which had lost >20% bodyweight compared to baseline, had to be sacrificed on day 6 according to predefined endpoints. As allodynia is a frequent symptom of chemotherapy-induced polyneuropathy, the mechanical withdrawal threshold was measured at various time points after suramin application. Suramin treated mice showed a significant decrease in the mechanical withdrawal threshold compared to vehicle-injected mice (Kruskal-Wallis test, *p* < 0.0001 and *p* = 0.0296 on day 8 and 13, respectively; Figure 1C), indicative of increased sensitivity to mechanical stimuli due to suramin treatment. In the rotarod test we also observed a mild deficit in locomotor function in suramin treated mice compared to control animals, which reached significance on day 8 (repeated measures 2-way ANOVA, *p* = 0.0022; Figure 1D). As the rotarod test measures a complex behavior which is influenced by motor performance but also coordination and sensory function, the observed decline in performance could be mediated by sensory neuropathy as well as motor neuropathy. Given the normal behavior of suramin treated animals in their home cage, the small effect size and our observations regarding the sensory nervous system, the effect of suramin on rotarod performance is likely dominated by sensory neuropathy. Electrophysiological assessment showed that suramin application led to a significant decline in the sensory nerve action potential amplitude (repeated measures 2-way ANOVA, *p* = 0.0158; Figure 1E) as well as the sensory nerve conduction velocity (Kruskal-Wallis, *p* = 0.0329 and *p* = 0.0008 on days 8 and 13, respectively; Figure 1F). In conclusion, our data provide evidence that a single injection of 250 mg/kg bodyweight suramin in C57Bl/6 mice is sufficient to induce a predominantly sensory axonal-demyelinating polyneuropathy.

### 2.2. Effects of Suramin on Cell Viability and Calcium Homeostasis in Dorsal Root Ganglia Neurons 

**Figure 2:**
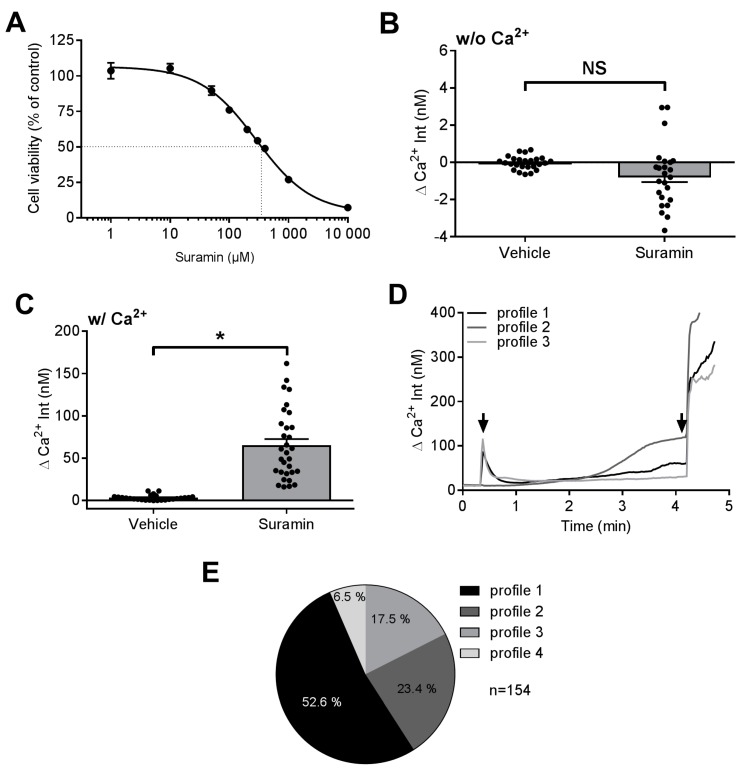
Effects of suramin on cell viability and intracellular calcium in dorsal root ganglia neurons. (**A**) Increasing suramin concentrations between 10 and 10,000 µM led to a dose-dependent decrease of cell viability with a calculated IC_50_ of 283 µM (non-linear regression analysis); (**B**) DRGN cultured in Ca^2+^ free buffer did not show alterations of intracellular Ca^2+^ levels after exposure with 1 mM suramin (24/9), whereas (**C**) increased intracellular Ca^2+^ levels could be observed when DRGN were incubated in Ca^2+^ containing medium and treated with 1 mM suramin (26/5); (**D**) Representative Ca^2+^ measurements of suramin treated DRGN: DRGN reacted to 1 mM suramin exposure with a transient Ca^2+^ increase followed by a slow increase (profile 1, black solid line), steady Ca^2+^ increase (profile 2, dark grey solid line) or a transient Ca^2+^ increase only (profile 3, light grey solid line). First arrow marks addition of 1 mM suramin, second arrow represents addition of 5 µM ionomycine (internal positive control); (**E**) Percentages of DRGN showing the different profiles of Ca^2+^ levels in response to 1 mM suramin exposure. * *p* < 0.05.

We have previously shown that some cytostatic agents cause alterations in intracellular calcium (Ca^2+^) homeostasis in dorsal root ganglia neurons (DRGN) and thus contribute to the development of chemotherapy-induced polyneuropathy [19,20,23,24]. In a first step, we were interested in the toxicity of suramin towards DRGN. We measured cell viability of DRGN in response to increasing concentrations of suramin and observed marked toxicity above 300 µM suramin concentrations. The calculated half maximal inhibitory concentration (IC_50_) of 22–24 h (hour) suramin treatment was 283 µM (non-linear regression analysis, 95% confidence interval 226–355 µM) in DRGN (Figure 2A). For the following experiments studying cell viability we used a dose of 400 µM. Next, we were interested in the effects suramin might exhibit on intracellular Ca^2+^ homeostasis. Sun and Windebank could previously show a calcium (Ca^2+^) influx into DRGN under “acute” suramin treatment [18]. In order to quantify and localize the impact of suramin on intraneuronal Ca^2+^ homeostasis, Ca^2+^ imaging experiments were performed using the fluorescent Ca^2+^ indicator dye fura-2. A higher dose of 1 mM suramin was chosen in order to ensure robust activation of potential receptors in the plasma membrane of DRGN despite of the short incubation time. First, we monitored Ca^2+^ levels in DRGN cultured in Ca^2+^ free medium, which did not result in any changes of intracellular Ca^2+^ levels (unpaired two-sided *t*-test; Figure 2B). However, when DRGN were cultured in Ca^2+^ containing medium, we observed a strong increase of intracellular Ca^2+^ levels (SUR 65.2 nM ± 7.4 nM, VEH 3.2 nM ± 0.6 nM), which is in line with previous findings (Mann-Whitney-U test, *p* < 0.0001; Figure 2C). These findings suggest that suramin exposure leads to an influx of Ca^2+^ from the extracellular space into the cytosol of DRGN via the plasma membrane. Intriguingly, the addition of suramin to DRGN cultures in Ca^2+^ containing imaging buffer caused different profiles of Ca^2+^ influx (Figure 2D): Most DRGN showed a rapid but transient Ca^2+^ influx followed by a slow Ca^2+^ increase (profile 1, 54%), others reacted with a slow but steady Ca^2+^ increase (profile 2, 23%) whereas some cells showed a short but transient Ca^2+^ increase only (profile 3, 18%). A smaller group of cells did not show any rise in intracellular Ca^2+^ levels after suramin treatment (profile 4, 7%) (Figure 2E).

### 2.3. Effects of Various Inhibitors of Plasmamembrane Channels on Suramin-Induced Neurotoxicity

Our data obtained from Ca^2+^ imaging indicated, that suramin treatment leads to an increase of intracellular Ca^2+^ levels via a Ca^2+^ influx across the plasma membrane. In a next step, we aimed to identify specific Ca^2+^ channels in the plasma membrane which are modulated by suramin. It was previously shown that nimodipine, an inhibitor of L-Type VGCC, has a protective effect on axonal outgrowth in suramin treated DRGN [18]. Based on our dose-response curve, we used suramin concentrations of 400 µM as a clinically relevant concentration that reproducibly led to a decrease of cell viability down to 52% ± 4% of vehicle treatment. We measured cell viability of suramin treated cells with and without co-incubation with various inhibitors of VGCC. Because of the complex chemical structure of suramin, we hypothesized that suramin could also induce Ca^2+^ influx into DRGN via transient-receptor-potential- (TRP-) channels. These are cation-conducting channels, which play an important role in sensory perception. A variety of TRP-channels subgroups are found in the plasma membrane of DRGN. At first, we screened for potential neuroprotective effects of different VGCC and TRP-channel inhibitors after co-incubation with 400 µM suramin using the MTT-assay. In case of neuroprotective effects on cell viability, calcium imaging was performed with those previously identified inhibitors. Table 1 summarizes the results of cell viability measurements of DRGN treated with 400 µM suramin in the presence of different VGCC- and TRP-inhibitors. 

**Figure 3:**
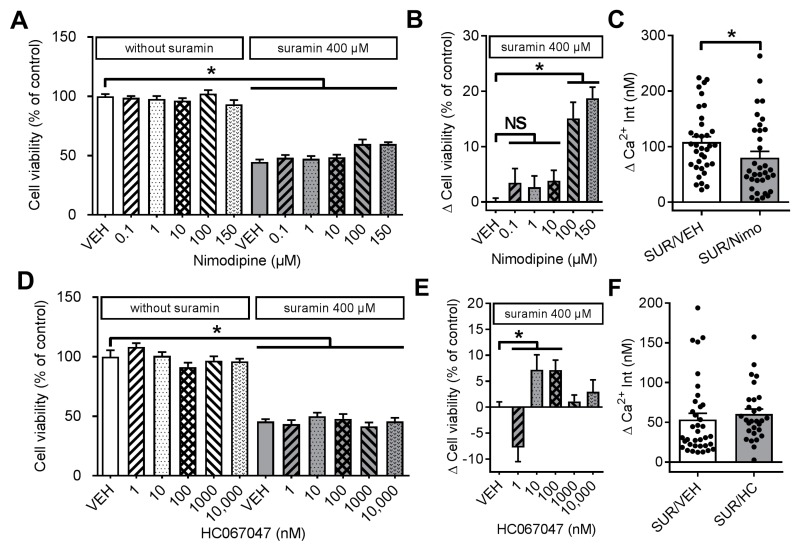
Effects of nimodipine and HC067047 on cell viability and intracellular Ca^2+^ in DRGN. (**A**) Treatment of DRGN for 24 h with suramin (400 µM) significantly decreased cell viability. Co-incubation with L-type VGCC nimodipine had a dose-dependent protective effect on cell viability; (**B**) Nimodipine (Nimo) concentrations of 100 and 150 µM significantly increased cell viability of DRGN in the presence of suramin; (**C**) Nimodipine (150 µM) reduced suramin-induced Ca^2+^ influx in DRGN, but could not fully prevent it; (**D**) Cell viability measured after treatment of DRGN with the increasing concentrations of the TRPV4 inhibitor HC067047 with and without suramin; (**E**) We observed a small but significant increase of cell viability when DRGN were co-incubated with HC067047 (10–100 nM), while (**F**) HC067047 (HC, 100 nM) did not improve suramin-induced Ca^2+^ influx into DRGN (18-42/3-7). * *p* < 0.05.

We observed a dose-dependent increase of cell viability, when DRGN exposed to 400 µM suramin were co-incubated with nimodipine (Figure 3A). This effect reached statistical significance for nimodipine concentrations of 100 µM and 150 µM, but could not restore impaired cell viability to control levels (1-way ANOVA, *p* < 0.0001; Figure 3B). We additionally measured intracellular Ca^2+^ levels of suramin treated DRGN under co-incubation with nimodipine. In line with the findings of the cell viability assays, we observed a significantly reduced Ca^2+^ influx in DRGN under co-incubation with suramin and nimodipine, compared to DRGN treated with suramin and the corresponding vehicle (Mann-Whitney-*U* test, *p* = 0.0229; *n* = 35 SUR/VEH, *n* = 34 SUR/Nimo; Figure 3C). Again, nimodipine was not able to prevent suramin-induced Ca^2+^ influx completely.

Efonidipine, another L-Type channel antagonist, did not exert protective effects on suramin-induced cell death. However, data on efonidipine is hard to interpret as increasing doses of efonidipine in the absence of suramin per se exert toxic effects on cultured DRGN with a significantly lower cell viability (1-way ANOVA, *p* = 0.0388 (10 µM) and *p* < 0.0001 (50 µM), respectively). The same problem was observed for the unselective VGCC and TRP inhibitor Ruthenium Red (1-way ANOVA, *p* = 0.0005; Supplementary Materials Table S1 and Supplementary Materials Figure S1). Additional VGCC antagonists did not show any effects on cell viability of DRGN after suramin treatment (Table 1). 

Next, we investigated various TRP channel inhibitors regarding their neuroprotective effects under suramin exposure. We only observed a small but significant protective effect on cell viability when DRGN were co-incubated with concentrations of 10–100 nM of the selective TRPV4 antagonist HC067047 (1-way ANOVA, *p* = 0.0294 (10 nM) and *p* = 0.0403 (100 nM), respectively; Figure 3D,E). However, HC067047 did not change Ca^2+^ influx due to suramin treatment (Mann-Whitney-*U* test; Figure 3F). Other TRP antagonists had no effects on cell viability after suramin exposure and measurements of intracellular Ca^2+^ levels of suramin treated DRGN did not show any alterations when DRGN were co-incubated with TRP antagonists.

### 2.4. Downstream Apoptotic Pathways and Experimental Limitations

In order to investigate apoptotic pathways further downstream of Ca^2+^ influx, we measured caspase activity after suramin treatment of DRGN. Surprisingly, we did not detect any protective effects of caspase-3/7 or calpain inhibition on cell viability when DRGN were co-incubated with suramin and the respective inhibitors (Supplementary Materials Table S2). We also observed a significant decrease of caspase-3/7 activity after 0, 6 and 12 h suramin treatment (Supplementary Materials Figure S2A). It was previously described in the Jurkat cell line that suramin itself can inhibit caspase-3/7 activity [25]. However, we also observed a significant increase in caspase-3/7 activity after 24 h suramin incubation. This observation is difficult to reconcile with the initially observed decrease of the luminescenct signal and we hypothesized that the luminescent assay signal might be quenched in the presence of suramin. Indeed, when we added suramin directly before luminescent signal detection, we also observed a significant decrease in measured caspase activity (Supplementary Materials Figure S2B). We therefore suggest that suramin either binds under a chemical reaction directly to the luminophore and thereby alters photon emission or it potentially absorbs photons emitted by the luminophore. Nevertheless, the significantly increased caspase activity after 24 h of suramin incubation suggests caspase activation by suramin, even considering the experimental limitations of the assay. We additionally performed immunhistochemical staining in order to verify our hypothesis. During those experiments, suramin interaction with cell culture coated dishes was observed. DRGN did not adhere to cell culture dishes under suramin treatment when washed according to the staining protocol. All cell culture surfaces were coated with a well-established protocol for DRGN cultures using poly-l-lysin and laminin. Prighozinha et al. described Matrigel dissolvement by suramin, which is of interest as Matrigel consists of 61% laminin. It was hypothesized that negatively charged sulfate groups would account for laminin binding and Matrigel dissolvement [26]. In line with these findings suramin was previously described to act as a heparan-sulfate analogue and antimetastic tumor activity of suramin was linked to this mode of action. Furthermore, suramin inhibition of binding of extracellular tumorcell-glycocalyx to the extracellular matrixprotein laminin was proposed to prevent tumor cell migration [27,28,29]. The same mechanism could be responsible for reduced binding activity of DRGN to laminin-coated surfaces. This means that suramin could inhibit binding of extracellular glycocalyx of DRGN to laminin molecules of the coated cell culture dish.

## 3. Discussion

The objective of this study was to elucidate the pathomechanism underlying suramin-induced neurotoxicity and its potential mechanistic link to neuronal Ca^2+^ dyshomeostasis. Intriguingly, different chemotherapeutic agents were found to disturb intracellular Ca^2+^ levels in neurons resulting in neuronal apoptosis. One example is paclitaxel, a chemotherapeutic agent causing a peripheral sensory neuropathy, which induces a dyshomeostasis of intracellular Ca^2+^ in DRGN [20,23]. It was proposed that the phosphoinositide pathway is involved and causes Ca^2+^ release from the endoplasmic reticulum under treatment with paclitaxel. Salinomycin, another experimental chemotherapeutic agent, was found to cause increased intracellular Ca^2+^ levels by its ionophoric capacity [19]. Furthermore, caspases and Ca^2+^ activated proteases such as calpains were shown to beinvolved in Ca^2+^-induced apoptosis. Caspase 12, a cytosolic caspase bound to the endoplasmic reticulum, also plays a role in Ca^2+^ triggered apoptosis [30]. In the context of suramin, Sun and Windebank proposed that Ca^2+^ influx into the cytosol would be mediated by channels in the plasma membrane [18]. Our Ca^2+^ imaging experiments confirm this hypothesis. In our study, we observed that suramin induces an increase of intracellular Ca^2+^ in DRGN only in Ca^2+^ containing buffer, whereas intracellular Ca^2+^ levels were not affected when DRGN were cultured in Ca^2+^ free buffer. This implies that suramin activates Ca^2+^ conducting channels in the plasma membrane causing a Ca^2+^ influx via the plasma membrane into the cytosol. Furthermore, it was shown that the L-type VGCC-inhibitor nimodipine can restore neuronal survival and axonal outgrowth after suramin treatment [18]. In our experiments, we also observed that nimodipine improves cell viability compared to suramin/vehicle co-treatment. Our data however also suggest that nimodipine cannot fully reverse suramin’s neurotoxic effects. In line with these findings, suramin-induced Ca^2+^ influx was significantly reduced when DRGN were co-incubated with nimodipine, but intracellular Ca^2+^ levels still remained elevated compared to control levels. Although these observations suggest that, the effect of nimodipine depends at least partially on inhibition of L-type VGCC, we observed robust neuroprotective effects only at comparatively high nimodipine concentrations above 100 µM. This concentration is at least an order of magnitude higher compared to the concentration of 1–10 µM typically used in in vitro studies to block L-type VGCC (e.g., [31,32]), suggesting the possibility of additional molecular mechanisms. Among others, nimodipine was shown to interact with multidrug resistance transporters such as P-glycoprotein [33] and at higher concentrations with voltage dependent potassium channels [34]. To further investigate this aspect we used efonidipine as another specific inhibitor of L-type VGCC and ruthenium red as a broad range inhibitor of Ca^2+^ channels in the plasma membrane (including VGCC and TRP). Unfortunately, these substances were themselves toxic, making the results hard to interpret. 

Another possible source for the Ca^2+^ influx across the plasma membrane are TRP channels. We tested a number of TRP channel inhibitors targeting TRP channel 3 (TRPC3), TRP channel subfamily A (TRPA1), subfamily V (TRPV4) and subfamily M inhibitors, of which only the TRPV4 inhibitor yielded a slightly positive effect on suramin-induced cell death. This is an interesting finding, as TRPV4 has been shown to play an important role in neuropathic pain caused by paclitaxel [35]. Given the diversity of TRP channels alone however, one limitation of the present work is that only a small subset of potentially relevant ion channels was studied. A logical next step for future experiments would be to repeat our experimental paradigm combined with whole cell patch clamp experiments to further characterize the mechanisms underlying Ca^2+^ influx. Subsequently additional inhibitors should be evaluated.

DRGN exhibited different responses to suramin in terms of their intracellular Ca^2+^ levels. We hypothesize that suramin might stimulate different ion channels or receptors in the plasma membrane by its unspecific binding properties. It should also be noted that DRGN differ in receptor and ion channel status. VGCC expression patterns vary especially between small, medium and large diameter neurons [36,37,38]. Also TRP-channel subtype expression was found to be variable in different DRGN [39]. Therefore, the unspecific binding properties of suramin on the one hand and varying receptor/ion channel status on DRGN on the other hand could lead to different reaction patterns of Ca^2+^ influx in DRGN after suramin treatment. This observation might explain the small effect size and heterogenous results observed in case of TRPV4 inhibition and the lack of changes observed in Ca^2+^ imaging experiments. This experimental hurdle could be overcome by studying for instance only TRPV4 positive DRGN. 

Increasing intracellular concentrations of Ca^2+^ in the cytosol can trigger apoptotic processes by initiating different signaling pathways. Most frequently caspases and calpains are involved. Unexpectedly, we did not see any positive effects of caspase or calpain inhibition regarding cell viability after suramin treatment, which is in contrast to previous results of Sun and Windebank, who observed protective effects of calpain inhibition. Future studies should expand on this aspect as it suggests a more complex mechanism of cell death.

Taken together, our data suggest a link between suramin-induced neurotoxicity and Ca^2+^ dyshomeostasis in DRGN and we could demonstrate that suramin treatment leads to a predominantly sensory axonal-demyelinating neuropathy in mice. However, it has also become clear in our experiments that suramin is a complex molecule and a whole array of cellular effects induced by suramin were described in the past (reviewed by [1]) making it a “dirty” drug. A number of mechanisms may thus contribute to suramin-induced neurotoxicity in the peripheral nervous system including but not limited to Ca^2+^ dyshomeostasis, interference with nerve growth factor signaling [16,17], glycolipid homeostasis [40], inhibition of P2X/P2Y purinoreceptors (reviewed by [41]) and mitochondrial toxicity [42]. As suramin acts as inhibitor of calcium conducting P2X purinoreceptors it seems that this target does not play a role in suramin-induced calcium influx in DRGN [43]. L-type Ca^2+^ channels seem to account only for a partial increase in intracellular Ca^2+^ levels after suramin treatment. Nevertheless, they present a potentially interesting molecular target as VGCC inhibitors such as nimodipine have already been approved for clinical use and could theoretically be easily translated to clinical application. Additional animal studies are however needed to prove a beneficial effect of VGCC inhibition in chemotherapy-induced polyneuropathy. Further ion channels in the plasma membrane also seem to mediate suramin-induced Ca^2+^ influx. One of those targets could be TRP channels, which have already been linked to chemotherapy-induced polyneuropathy in other antineoplastic substances such as paclitaxel. 

In summary, given suramin’s complex biological and chemical characteristics and proven neurotoxicity, developing a neuroprotective co-treatment may help to overcome suramin’s translational challenge and enable a broader clinical use of this drug.

## 4. Materials and Methods

### 4.1. In Vivo

#### 4.1.1. Animal Housing, Sample Sizes and Methods of Randomization and Blinding

We used 20 nine-week old male C57Bl/6 mice from Charles River (Sulzfeld, Germany) for the experiment. All procedures had been previously approved by the State Office for Health and Social Affairs (Landesamt für Gesundheit und Soziales (LaGeSo), Berlin, Germany, Reg.-Nr: G0092-10) and conformed to animal welfare guidelines. Mice were housed in groups of five in an enriched environment and a 12-hour light/dark cycle (7 a.m.–7 p.m.) with unlimited access to food and water. The a priori calculated sample size was 10 mice/group, based on previously observed effect sizes of electrophysiological parameters [21], a power of 0.8 and an alpha-error of 0.05. Sample size calculation was done using G*Power statistical software (Heinrich Heine Universität Düsseldorf, Düsseldorf, Germany) [44]. Mice were randomized to one of two groups (SUR vs. VEH) using an online randomization tool (GraphPad Software, La Jolla, CA, USA; https://www.graphpad.com/quickcalcs/randomize1.cfm). Behavior experiments were conducted by a blind investigator. 

#### 4.1.2. Drug Preparation and Injection

Suramin (Tocris, Bioscience, Bristol, UK) was dissolved in Aqua dest. to a concentration of 50 mg/mL and diluted in sterile 0.9% saline to a final concentration of 25 mg/mL and injected once intraperitoneally at 10 µL/g bodyweight. As vehicle treatment, Aqua dest. was diluted 1:1 in 0.9% sterile saline and injected once intraperitoneally at 10 µL/g bodyweight. 

#### 4.1.3. Behavior Analysis

Mice were handled for five consecutive days before behavior experiments to ensure familiarization to the investigator and to minimize stress and anxiety. All behavior experiments were done in a dedicated laboratory with soundproof chambers during 10 a.m. and 4 p.m. The general wellbeing of the mice was checked daily and their weight recorded. The mechanical withdrawal threshold was obtained using the von Frey filament test as previously described [21,45]. In brief: a hand-held force transducer was fitted with a 0.5 mm^2^ polypropylene tip. Mice were placed in a clear inverted plastic cage on a wire-mesh floor. Trained investigators applied increasing pressure to the center of the hind paws until a clear withdrawal response was evoked. If the animal did not exhibit a clear withdrawal response within 5 s the trial was deemed invalid and was repeated. The withdrawal threshold in grams (g) was automatically measured by the device (IITC, Woodland Hills, CA, USA). The maximum applied force was 10 g. For each time point, 5 trials were averaged. Locomotor function was measured with the rotarod test as previously described [21,45]. Mice were placed on a rotating rod (TSE Systems, Bad Homburg, Germany), which gradually increased in speed from 4 to 40 rpm over the course of 300 s. The latency for the mice to fall off the rod was automatically measured by a floor sensor. Mice were trained in the task for four consecutive days prior to baseline measurement. For each time point, three trials for each animal were averaged. 

#### 4.1.4. Nerve Conduction Studies

Mice were anaesthetized with isoflurane 1.5%/50% O_2_ (volume/volume) and placed on a heating pad with a constant temperature of 37.5 °C (Harvard Apparatus, Holliston, MA, USA). Stimulating needle electrodes were placed at the base of the tail with the recording electrodes approx. 5 cm distal. A ground electrode was place in the middle. Single stimuli were applied to determine supramaximal level and afterwards 50 serial supramaximal stimuli were averaged to obtain the sensory nerve action potential (SNAP) amplitudes and NCV [21,45].

### 4.2. In Vitro

#### 4.2.1. DRGN Cell Culture

DRGN cultures were obtained from P 0–3 Wistar rat neonates as described before [23]. Cells were plated on poly-l-lysin and laminin coated 96-well plates for cell viability measurements and on cover slips with the same coating for Ca^2+^ imaging experiments. DRGN cultures were incubated overnight at 37 °C in a 95% air/5% CO_2_ humidified atmosphere before experimental use. Neurobasal A medium supplemented with B-27 (Life Technologies GmbH, Darmstadt, Germany), 0.5 mM glutamine, fresh nerve growth factor (10 ng/mL) was used for cell maintenance. 

#### 4.2.2. Calcium Imaging

Cells were mounted with Fura-2 AM 5 µM (Life Technologies GmbH, Darmstadt, Germany) for 30 min at 37 °C in a standard HEPES buffered solution: 130 mM NaCl/4.7 mM KCl/1 mM MgSO_4_/1.2 mM KH_2_PO_4_/1.3 mM CaCl_2_/20 mM Hepes/5 mM glucose (pH 7.4)/0.02% pluronic F-127 (Life Technologies GmbH, Darmstadt, Deutschland). After incubation cell cultures were washed with standard HEPES buffered solution and coverslips were placed in a Olympus IX 81 microscope equipped with a Uplan FLN oil objective 40×/1.13 (Olympus Corporation, Tokyo, Japan). For calcium imaging experiments in Ca^2+^ free extracellular medium a standard HEPES solution without Ca^2+^ was used: 130 mM NaCl/4.7 mM KCl/2.3 mM MgSO_4_/1.2 mM KH_2_PO_4_/10 mM EDTA/20 mM Hepes/5 mM glucose, pH 7.4. For experiments with ion channel inhibitors DRGN cultures were preincubated in standard HEPES buffered solution with the respective channel blocker for 5 min before Ca^2+^ levels were measured. Fluorescence signals were detected by a cooled CCD-camera and data was processed on a computer using Olympus xcellence imaging software. Intracellular Ca^2+^ concentrations [(Ca^2+^) int] (nM) were calculated using ratios of F340/F380 after background subtraction with the following equation: [Ca^2+^]int (nM) = *K*d*Q*(*R* − *R*_min_)/(*R*_max_ − *R*), where *K*d is the dissociation constant of Fura-2 for Ca^2+^ at room temperature (225 nM); Q is the fluorescence ratio of the emission intensity excited by 380 nm in the absence of Ca^2+^ to that during the presence of saturating Ca^2+^; and *R*_min_ and *R*_max_ are the minimal or maximal fluorescence ratios, respectively. Values for *R*_min_ and *R*_max_ were derived from in situ calibration using Ca^2+^ free HEPES buffered standard solution with 10 µM ionomycin for *R*_min_ values and HEPES buffered standard solution with CaCl_2_ for *R*_max_ values. Intracellular free Ca^2+^ concentrations were measured over 4.37 min adding 1 µM suramin or VEH (Aq. dest.) after 20 s and 10 µM ionomycin after 4.15 min (internal control). Cells with an increase of <200 nM [Ca^2+^ int.] (Δ) in HEPES buffered solution with Ca^2+^ or <50 nM [Ca^2+^ int.] (Δ) in HEPES buffered solution without Ca^2+^ were excluded from the data analysis.

#### 4.2.3. MTT-Assay

DRGN cultures plated in 96 well plates were incubated with suramin and ion channel inhibitors (VGCC and TRP-channels) and respective controls for 24 h at 37 °C in a 95% air/5% CO_2_ humidified atmosphere. MTT was added to each well in a ratio of 1:10 and cell cultures were reincubated for 30 min at 37 °C in a 95% air/5% CO_2_ humidified atmosphere. Reaction was stopped using SDS in a ratio of 1:1. After cell dissolution overnight, absorption values were gained at a wavelength of 550 nm, lamp energy of 10,000 during 0.2 s in a microplate reader (Tristar LB941 Multimode Microplate Reader Berthold Technologies GmbH and Co. KG, Bad Wildbad, Germany). Values were background subtracted and standardized to percentage of control. For coincubation with suramin and ion channel inhibitors a second standardization was used and the mean difference suramin plus inhibitor to suramin plus vehicle treatment (ΔSUR/VEH) was calculated. 

#### 4.2.4. Caspase Assay

Caspase activity was assessed using the Promega *Caspase-Glo*^®^ *3/7 Assay kit* (Promega, Mannheim, Germany) according to the manufacturer’s protocol as described [46].

### 4.3. Statistical Analysis and Exclusion Criteria

The manuscript was prepared in accordance with ARRIVE guidelines [47]. Data is presented as mean ± SEM with individual values depicted for in vivo studies. For MTT assay experiments, data was obtained from *n* = 3–6 individual experiments (biological replicates) with *n* = 3 technical replicates. For Ca^2+^ imaging experiments (*n*/*N*) describes the number of cells studied (*n*) in (*N*) independent cultures. Statistical analysis was performed using Prism v6.0 (GraphPad Software, La Jolla, CA, USA). Gaussian distribution of data was checked prior to statistical analysis using Shapiro-Wilk normality test. Normally distributed data was analyzed using unpaired two-sided *t*-tests (two groups), one-way ANOVA with Sidak post hoc test (≥three groups) or (repeated measures) 2-way ANOVA (two variables). Not normally distributed data was analyzed with Mann-Whitney-*U* test (2 groups) or Kruskal-Wallis test with Dunn’s method (≥3 groups). *p* < 0.05 was considered statistically significant and is depicted by asterisk. Only statistical outliers that conformed with Peirce criterion were excluded from the dataset [48,49].

## Figures and Tables

**Table 1:** Summary of observed cell viability of DRGN after suramin treatment in the presence of different inhibitors.

**Substance**	**Nimodipine (L-Type VGCC Inhibitor)**					**A 967079 (TRPA1-Inhibitor)**				
SUR 400 µM +	0.1 µM	1 µM	10 µM	100 µM	150 µM	1 nM	10 nM	100 nM	1 µM	10 µM
Change attributable to intervention (Δ% of SUR/VEH)	+3.5 ±2.6	+2.7 ±2.0	+3.9 ±1.9	+15.1 * ±2.9	+18.7 * ±2.1	+0.2 ±2.3	+7.1 ±3.5	+3.0 ±2.4	+2.6 ±1.2	+2.8 ±4.8
**Substance**	**Efonidipine (L-Type VGCC Inhibitor)**					**HC 067047 (TRPV4 Inhibitor)**				
SUR 400 µM +	0.1 µM	1 µM	10 µM	50 µM		1 nM	10 nM	100 nM	1 µM	10 µM
Change attributable to intervention (Δ% of SUR/VEH)	+3.5 ±2.7	+0.5 ±2.6	+0.6 ±2.7	−1.6 ±3.2		−7.5 * ±3.0	+7.2 * ±2.9	+7.1 * ±2.0	+1.1 ±1.2	+2.9 ±2.3
**Substance**	**Ruthenium Red (unselective including VGCC and TRP Inhibition)**					**Pyr 3 (TRPC3 Inhibitor)**				
SUR 400 µM +	10 nM	100 nM	1 µM	10 µM		1 nM	10 nM	100 nM	1 µM	10 µM
Change attributable to intervention (Δ% of SUR/VEH)	+8.1 ±4.2	+4.4 ±1.6	+7.6 ±3.9	−1.5 ±2.8		+0.2 ±2.5	−3.4 ±4.2	−1.3 ±3.8	−2.8 ±3.0	−2.6 ±2.5
**Substance**	**SNX 482 (R-Type VGCC Inhibitor)**					**Ononetin (TRPM3 Inhibitor)**				
SUR 400 µM +	2 nM	20 nM	200 nM			3 nM	30 nM	300 nM	3 µM	30 µM
Change attributable to intervention (Δ% of SUR/VEH)	−3.2 ±1.8	−2.2 ±3.0	+2.1 ±2.3			−5.9 ±2.6	+2.5 ±2.1	−2.2 ±2.4	−2.7 ±3.3	−1.9 ±2.7
**Substance**	**Ω-Conotoxin MVIIC (N-, P-, Q-Type VGCC Inh)**									
SUR 400 µM +	1 nM	10 nM	100 nM	1 µM						
Change attributable to intervention (Δ% of SUR/VEH)	−2.2 ±1.8	−2.0 ±2.6	+1.9 ±1.6	+1.9 ±3.3						

* Statistically significant (*p* < 0.05).

## Supplementary Materials

The Supplementary Materials are available online.

## Abbreviations

Ca^2+^	calcium
DRGN	dorsal root ganglia neurons
VGCC	voltage-gated calcium channels
TRP	transient-receptor-potential
