# Supplementary material for: Suramin-Induced Neurotoxicity: Preclinical Models and Neuroprotective Strategies
Source: Molecules. 2018 Feb 7;23(2):346. doi: 10.3390/molecules23020346 (PMC6017835; doi:10.3390/molecules23020346)
Supplement: Supplementary file 1 [file molecules-23-00346-s001.pdf]

Supplemental Information

Table S1. Effects of different inhibitors on cell viability of DRGN.

**Substance**	**Nimodipine (L-Type VGCC Inhibitor)**					**A 967079 (TRPA1-Inhibitor)**				
	0.1µM	1µM	10µM	100µM	150µM	1nM	10nM	100nM	1µM	10µM
**% of VEH**	98,8 ± 1,5	97,8 ± 2,5	96,4 ± 2,1	102,3 ± 3,0	93,3 ± 4,2	112 ± 2,5	100,9 ± 3,1	101,2 ± 2,1	100,6 ±2,3	95,3 ± 4,2
**Substance**	**Efonidipine (L-Type VGCC Inhibitor)**					**HC 067047 (TRPV4 Inhibitor)**				
	0.1µM	1µM	10µM	50µM		1nM	10nM	100nM	1µM	10µM
**% of VEH**	93,2 ± 2,0	97,8 ± 1,4	90,1* ± 2,9	75,5* ± 3,0		105,7 ± 2,0	95,4 ± 3,0	89,7* ± 3,1	96,1 ± 2,5	91,2 ± 3,2
**Substance**	**Ruthenium Red (unselective including VGCC and TRP Inhibition)**					**Pyr 3 (TRPC3 Inhibitor)**				
	10nM	100nM	1µM	10µM		1nM	10nM	100nM	1µM	10µM
**% of VEH**	91,9 ± 4,3	98,9 ± 6,6	96,0 ± 4,0	76,5* ± 3,5		100,0 ± 3,1	106,9 ± 2,2	105,2 ± 4,5	106,0 ± 4,9	97,3 ± 3,8
**Substance**	**SNX 482 (R-Type VGCC Inhibitor)**					**Ononetin (TRPM3 Inhibitor)**				
	2nM	20nM	200nM			3nM	30nM	300nM	3µM	30µM
**% of VEH**	98,3 ± 3,9	102,1 ± 2,5	98,1 ± 4,4			100,0 ± 2,9	100,4 ± 3,4	98,9 ± 3,1	90,5 ± 4,5	92,4 ± 3,8
**Substance**	**Ω-Conotoxin MVIIC (N-,P-,Q-Type V GCC Inhibitor)**									
	1nM	10nM	100nM	1µM						
**% of VEH**	99,7 ± 3,4	95,1 ± 1,9	96,5 ± 2,5	97,6 ± 2,3						

* statistically significant (p<0.05)

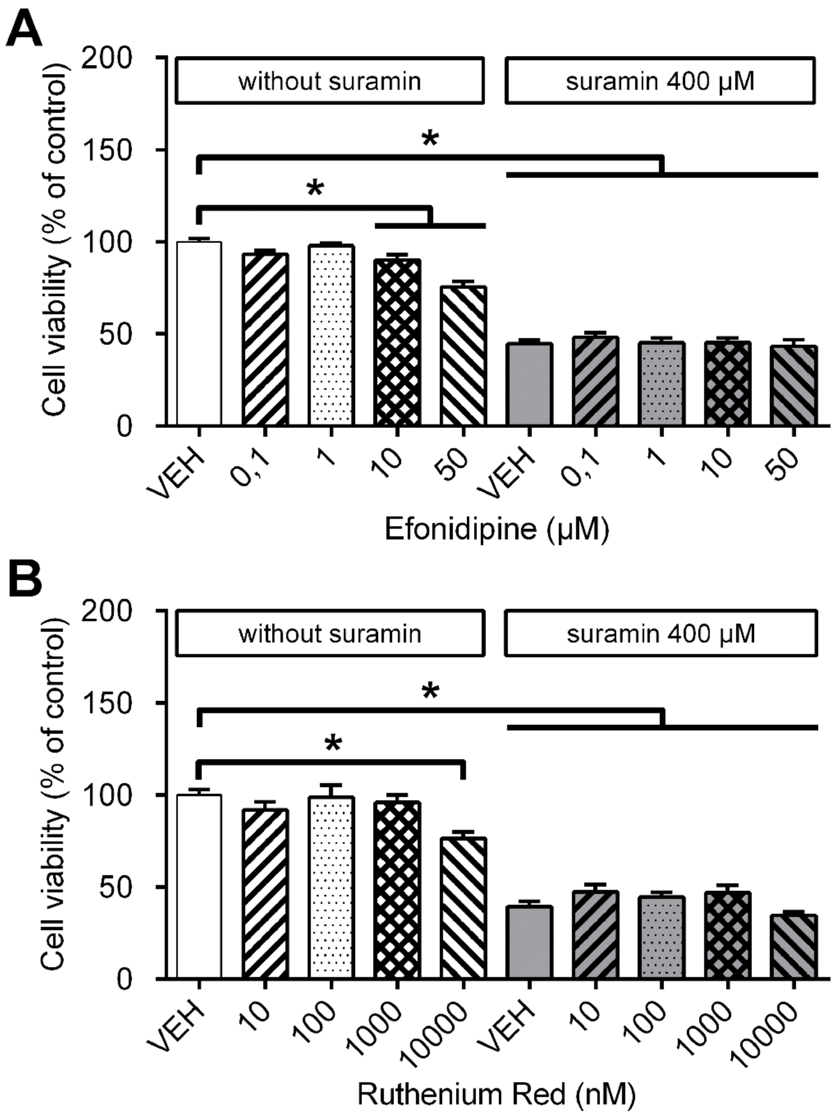

Figure S1. Effects of VGCC inhibitors on cell viability of DRGN.

(**a**) Cell viability of DRGN was markedly reduced after exposure to suramin and could not be restored by co-incubation with increasing concentrations of the L-type VGCC inhibitor Efonidipine. However, Efonidipine in concentrations ≥10 µM itself reduced cell viability . (**b**) Similar effects were found for the unselective VGCC and TRP inhibitor Ruthenium Red, which was also toxic in higher concentrations and had no influence on cell viability when co-incubated with suramin.

Table S2. Effects of calpain and caspase inhibitors on cell viability of DRGN

Substance	MDL 28170 (Calpain Inhibitor)					Ac DEVD CHO (Caspase-Inhibitor)	
VEH (H_2O)+	1nM	10nM	100nM	1µM	10µM	1µM	10µM
% of VEH	99,6 ±5,0	104,3 ±3,2	95,3 ±4,0	93,3 ±4,5	89,7 ±3,1	95,5 ±5,7	91,8 ±5,7
SUR 400 µM+	1nM	10nM	100nM	1µM	10µM	1µM	10µM
Δ% of SUR/VEH	-7,1 ±2,4	+2,0 ±3,6	-2,9 ±3,1	+5,3 ±4,0	+3,8 ±3,3	+6,0 ±3,2	+3,8 ±2,0

* statistically significant ($p<0.05$)

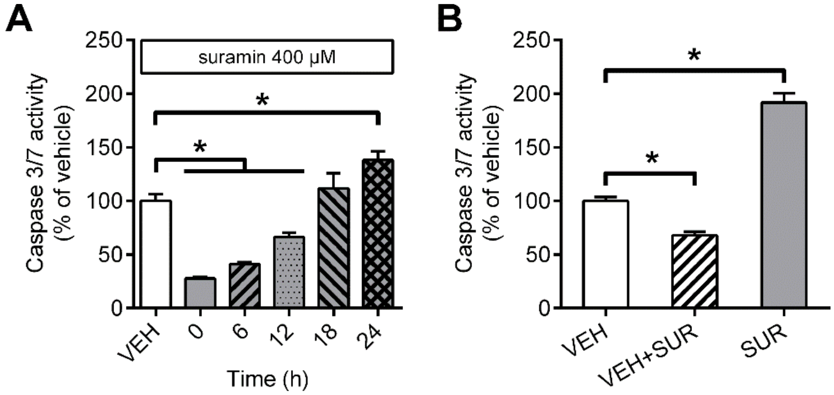

Figure S2: Caspase-3/7 activity of DRGN after suramin treatment.

(**a**) Caspase 3/7 activity was measured with a luminescent caspase assay. After incubation of DRGN cultures with suramin for 0, 6 and 12 h a significantly reduced luminescent signal was detected. However, the signal was significantly increased after suramin incubation for 24 h. (**b**) When DRGN were incubated with vehicle for 24 h and suramin was thereafter added to the culture immediately before luminescence signal detection (VEH+SUR), a decrease of caspase activity was observed, suggesting that suramin quenches the luminescence signal. Again, an increase in caspase-3/7 activity was observed when DRGN were treated with suramin for 24 h.
